# Book Reviews

**Published:** 1890-08

**Authors:** 


### SOCIETY TRANSACTIONS.

I. Transactions of the Medical Society of the State of New York. Eighty-fourth year. 1890. Edited for the Society by Frederic C. Curtis, M. D., Secretary. Standard octavo. Illustrated. Pp. viii.—490. Published by the Society. Philadelphia: William J. Dornan, Printer. 1890.

II. Transactions of the American Gynecological Society. Vol. XIV. For the year 1889. Small octavo. Pp. 480. Edited for the Society by by Joseph Taber Johnson, M. D, Secretary, Washingtou, D. C. Philadelphia: William J. Dornan. 1889.

III. Transactions of the Association of American Physicians. Fourth session, held at Washington, D. C., September 18, 19, and 20, 1889. Vol. IV. Standard octavo. Pp. xviii.—381. Printed for the Association. Philadelphia: William J. Dornan. 1889.

IV. Transactions of the Mississippi State Medical Association at the Twenty-second Annual Session, held at Jackson, April 17, 18, and 19, 1889. Published by the Association. Standard 8vo; pp. 188. Jackson: Clarion-Ledger Printing Establishment. 1889.

V. Transactions oe the Medical and Chirurgical Faculty of the State of Maryland, at its Ninety-first Annual Session, held at Baltimore, April, 1889. Paper, standard 8vo; pp. 264. Edited for the Faculty by G. Lane Taneyhill, M. D., Recording Secretary. Baltimore: Press of John B. Kurtz. 1889.

VI. Transactions of the Medical Association of the State of Missouri, at its Thirty-second Annual Session, held at Springfield, May 21 and 22, 1889. Paper, standard 8vo, pp. xiv.—218. St. Louis: Ev. E. Carreras, Printer. 1889.

VII. Transactions of the Medical Society of the State of North Carolina. Thirty-sixth Annual Session, held at Elizabeth City, April 16, 17, and 18, 1889. Paper, standard 8vo, pp. 164. Wilmington: Jackson & Bell, Printers. 1890.

VIII. Transactions of the Eighth Annual Meeting of the American Laryngological Association. Held in the City of Washington, D C, May 30 and 31, and June 1, 1889. 8vo, cloth, pp. iv.—150. New York: D. Appleton & Co. 1890.

I. The Transactions of the Medical Society of the State of New York, at its eighty-fourth annual session, held in Albany, N. Y., February 4, 5, and 6, 1890, comes to hand promptly, and it may justly be said of this volume that the society never published a better one. The scientific excellence of the book is attested by the contributions of such men as Keyes, Vander Veer, Bryant, Morris, Gibney, Chase, Corning, Granden, Currier, Coe, Fox, Bulkley, Goodwillie, Roe, and a host of others. The sound financial status of the Society is made apparent in the treasurer's report, and, taken altogether, this is one of the best volumes of society transactions we have seen for many a day. The Society has only to keep steadily on in its present lines of good work to command the respect of the entire medical world.

II. The Transactions of the American Gynecological Society, for 1889, presents itself in its usually attractive form. The volume is overflowing with interesting material that has already been largely noted in the journals. The paper by Dr. Garrigues, on the Use and Abuse of Antiseptic Injections in Obstetrical Practice, is well worth the price of the book, and the discussion thereon brings out the lights and shades of the picture in an ample manner. The next meeting of this Society will be held in Buffalo, September 16, 17, and 18, 1890.

III. The fourth annual volume of the Transactions of the Association of American Physicans contains nineteen papers of interest, upon which the discussions were able. The book, a modest standard octavo of xviii. — 381 pages, would make a valuable addition to any physician's library. It is edited by the secretary of the association, Dr. I. Minis Hays, and printed by Mr. William J. Dornan, of Philadelphia — two experienced men in their respective fields — and can be obtained upon application to either of these gentlemen.

IV. The Mississippi State Medical Association puts forth a neat volume of 188 pages, as the result of its work during its Twenty-second Annual Session. One of the very interesting papers in this book is by Dr. T. J. Crofford, of Memphis, entitled, A New Method of Performing Hysterectomy, in which he reports a case where he removed the entire organ, leaving no uterine tissue whatever. This is the method he advocates, and his case goes far to fortify his argument. Many of the other papers possess considerable interest, though we think it had been well if the discussions had also been published. Discussion is to a paper what a background is to a picture, and each is made to stand out in its best color when these are properly shaded.

V. The Medical and Chirurgical Faculty of Maryland is one of the oldest of the State Medical Societies, and always presents an interesting annual volume, this one for 1889 being no exception to the rule. It is organized into seven sections, viz.: Surgery; Practice of Medicine; Obstetrics and Gynecology; Materia Medica and

Chemistry; Anatomy, Physiology and Pathology; Microscopy, Micro-chemistry and Spectral Analysis; Ophthalmology, Otology and Laryngology. It seems to us that this is rather a cumbrous organization for a State society, particularly a sthe several chairmen of these sections are required to report on the progress in their departments, each in an annual address.

It is the custom of this Society to invite a prominent physician, usually from beyond the borders of the State, to deliver an "Annual Address," and we note that Dr. William Osler performed this duty in 1889, choosing for his subject The License to Practise. Among the many good points made in this address occurs the absurd advocacy of an elective State Board of Medical Examiners. It is to be hoped that never may the high office of State Medical Examiner be dragged into the purlieus of political intrigue and chicane.

This volume, though bound in paper, is edited by an experienced Secretary, Dr. G. Lane Taneyhill, and it is well printed.

VI. The State of Missouri has an active Medical Association, and its annual transactions contain some interesting and instructive papers. Those upon Abdominal Surgery indicate proficiency in that line of work, while many others are worthy of extended mention had we the space to give it. This book is also bound in paper — a draw-back to its preservation upon library shelves.

VII. The Medical Society of the State of North Carolina publishes its transactions in the *North Carolina Medical Journal*, whence this volume, bound in paper, containing 164 pages, is reprinted. This Society, also, does some excellent work, but its sub-division into sections seems to us a hindrance to the accomplishment of its best work. Dr. George Gillett Thomas was elected President at this (1889) meeting, and Dr. L. M. Hays was chosen Secretary.

VIII. The papers that enter into this volume of Transactions of the American Laryngological Association, have already appeared, we believe, in the *New York Medical Journal*, and, therefore, an extended comment on them is not necessary. We must, however, express our satisfaction at having them in this permanent form. Few, if any, of the special societies have accomplished better work than this one. We notice that its annual volume is always received with praise by those familiar with its line of work.

Dr. J. N. Mackenzie, of Baltimore, was elected President for the current year. This was a well-merited honor, and was most gracefully received.

Cyclopedia of the Diseases of Children, Medical and Surgical. The article written especially for this work by American, British and Canadian authors. Edited by John M. Keating, M. D. Vol. III.— 1371 pages. Illustrated. Philadelphia: J. B. Lippincott Co. 1890.

The third volume of this excellent work fully bears out the reputation of the preceding volumes. This volume is divided into four parts, devoted respectively to Diseases of the Digestive System, Diseases of the Genito-Urinary Organs and the Blood, Surgery, and Diseases of the Osseous System and the Joints.

In the short space at our command, it would be impossible to give an analytical review of the entire work, and it would be invidious to select special articles when each is the work of a master in his department. The article on Blood seemed entirely disconnected with the rest of the section in which it is included, but a footnote explains its appearance here instead of in Vol. II., where it more properly belonged, by stating that, as Dr. Griffith was asked to undertake its preparation at a late date, it was impossible to place it in that volume. The illustrations of the various articles are most excellent, those showing apparatus for correcting deformities being especially worthy of mention.

